# The Burden of Interstitial Lung Involvement in Rheumatoid Arthritis: Could Lung Ultrasound Have a Role in Its Detection? A Literature Review

**DOI:** 10.3390/diagnostics14131430

**Published:** 2024-07-04

**Authors:** Gemma Lepri, Milica Markovic, Silvia Bellando-Randone, Marco Sebastiani, Serena Guiducci

**Affiliations:** 1 Division of Rheumatology, AOU Careggi, Department of Experimental and Clinical Medicine, University of Florence, Via delle Oblate 4, 50141 Florence, Italy; 2 Department of Medicine and Surgery, University of Parma, 43126 Parma, Italy; 3 Rheumatology Unit, Hospital Guglielmo da Saliceto, 29121 Piacenza, Italy

**Keywords:** rheumatoid arthritis, interstitial lung disease, lung ultrasound, B-lines, high resolution computed tomography

## Abstract

Lung involvement represents a fearful complication in rheumatoid arthritis (RA), potentially involving all compartments of the pulmonary system. Regarding interstitial lung disease (ILD), the HRCT represents the gold standard technique for its diagnosis; however, the examination is burdened by radiation exposure and high costs. In addition, although some risk factors for ILD are known, no algorithms exist to know which patients to submit to HRCT and when. In this context, lung ultrasound (LUS) showed promising results for at least 10 years, demonstrating correlation with high resolution computed tomography (HRCT) findings in other rheumatic diseases. Here, LUS may represent a screening test providing additional information to clinical examination and pulmonary function tests. The data deriving from LUS experience in other rheumatic diseases could steer the future towards the use of this technique also in RA patients, and in this review, we report the most relevant literature regarding LUS in RA-ILD.

## 1. Introduction

In the last years, the definition of rheumatoid arthritis (RA) has radically changed. Before, it was considered an autoimmune, chronic and inflammatory joints disease with possible extra-articular manifestations [1], and in the last years, the concept of RA as a systemic disease has been established [2,3,4]. Of course, although the inflammatory process of the disease primarily damages joints, it often affects other organs, such as kidney, heart, lung, skin, digestive tract, eye and skin [4]. Above extra-articular involvements of RA, interstitial lung disease (ILD) represents one of the more common complications of RA, affecting about 20% of patients, with clinical significance in up to 10% of them [5,6]. Lung involvement in RA is associated with a significant increase in mortality and morbidity [7]. RA-ILD is more frequent in males, with a ratio of 2:1, and with onset within the fifth to sixth decade of life [8,9,10,11]. Smoking, seropositivity for rheumatoid factor or anti-citrullinated protein antibody (ACPA), disease duration and activity are the main risk factors associated with RA-ILD [12]. Increased risk of ILD in RA is also observed in patients with HLA-B54, HLA-DQ1B*0601 and HLA-B40 [13]. Furthermore, a gain-of-function promoter variant (rs35705950) in the mucin 5B (MUC5B) gene is observed to be associated with RA-ILD, more specifically with the usual interstitial pneumonia (UIP) pattern [14]. UIP represents the most common radiological and histopathologic pattern of RA-ILD (pooled prevalence of 46%) [15]. It is characterized by predominantly basal subpleural reticular abnormalities with honeycombing (HC), and traction bronchiectasis with a relative absence of ground-glass opacities (GGO) and air trapping on exhalation [16]. The clinical behavior of RA-ILD UIP pattern is variable, and some studies suggest it is similar to that of idiopathic pulmonary fibrosis (IPF), being associated with a high risk of acute exacerbations and poor response to treatment [17,18,19]. RA-ILD UIP pattern is associated with poorer prognosis and higher mortality compared to the nonspecific interstitial pneumonia (NSIP) pattern that, although it can also be found in patients with RA, is more common in other connective tissue diseases (CTDs), such as systemic sclerosis (SSc) [20,21,22]. Lung high resolution computed tomography (HRCT) represents the gold standard technique to diagnose ILD [23,24]. Pulmonary function tests (PFTs) are useful in the follow-up and at baseline; however, they alone are inadequate to diagnose ILD [24]. Lung auscultation may help in early identification of RA-ILD, while clinical evaluation remains inadequate to the early identification of ILD [25]. In fact, despite significant radiological involvement, RA-ILD patients may be asymptomatic or oligosymptomatic for a long time [9]. Chest HRCT is burdened by radiation exposure and high costs, and for this reason screening programs identifying which patients to submit to HRCT are widely warranted. In this context, a preliminary algorithm has been proposed considering the disease characteristics, serum Krebs von den Lungen-6 antigen (KL-6) levels and lung ultrasound (LUS) evaluation [26]. In fact, in the last decade ever greater attention has been focused on LUS. They have been proved to correlate with HRCT and to be able to identify ILD signs in other CTDs as in patients with early SSc, strongly suggesting LUS as a screening tool for ILD detection [27]. The LUS sign of lung interstitial involvement is represented by B-lines that are defined as discrete laser-like vertical hyperechoic reverberation artefacts that arise from the pleural line, extending to the bottom of the screen without fading, and move synchronously with the lung sliding [28,29,30]. Recently, using the SSc model, the OMERACT US working group performed a Delphi process and web-reliability exercise that defined B-lines and pleural line abnormalities as elementary lesions to assess; moreover, they confirmed the moderate to good reliability in detection of these irregularities in 80 video-clips of web-exercise [31]. Although a standardized scanning protocol and a scoring system are still missing, representing the next steps of the working group, these results confirmed the role of LUS in the detection of pulmonary anatomical changes related to ILD in SSc patients. The scientific enthusiasm for LUS in SSc-related ILD has also led to investigations of its possible role in other rheumatic diseases, such as RA. Our work aims to review the most relevant literature regarding LUS in RA-ILD, presenting the available evidence supporting its role.

## 2. Materials and Methods

Two investigators independently searched the databases PubMed and Scopus and screened the articles up to 1 March 2024. The search string included rheumatoid arthritis, lung ultrasound, B lines and pleural line irregularity. The articles not in English were not included. Letters, editorials, comments, meeting abstracts, case reports, systematic reviews or meta-analysis were excluded categories.

## 3. Review of Relevant Medical Literature

11 original articles [32,33,34,35,36,37,38,39,40,41,42] have been evaluated. Two of them analysed patients with different rheumatic diseases [33,35] (RA, SSc, systemic lupus erythematosus [SLE], Sjogren’s syndrome and dermatomyositis); the others only included RA patients with or without healthy controls. In addition to those 11 papers, we also mentioned a pre-publication of a Danish multicentre cross-sectional diagnostic test accuracy study—AURORA study that will evaluate diagnostic accuracy of LUS in RA-ILD [43].

In the reviewed studies, various scoring systems examining different lung intercostal spaces (LIS) have been used, such as all, 72, 14, 10 and 8 LIS (Table 1). In most of them, LUS is considered to be positive when the sum of B-lines was ≥5. In all studies, comparison with HRCT was evaluated.

In the two studies enrolling both RA and other CTDs patients, pathological US patterns were significantly more frequent in patients with ILD than in those without [33,35]. Moazedi-Fuerst et al. evaluated 45 patients (25 with RA, 14 with SSc and 6 with SLE) and 40 controls by transthoracic US and HRCT [34]. All patients with RA-ILD presented B-lines (in at least 1–5 regions) and pleural nodes (one or more pleural nodes in 1–5 areas) compared to 6% and 22%, respectively, of RA patients without ILD. A pleural thickness (>3 mm) was reported in 86% of RA patients with ILD and in 11% of those without ILD. Interestingly, RA-ILD patients presented a significant higher incidence of pleural nodes than SSc and SLE patients with ILD. The study was limited by the small number of enrolled patients; however, it suggested the potential role of LUS as a non-invasive technique to assess ILD in rheumatic patients [33]. The other study enrolling patients with different diseases (19 with SSc, 8 with RA, 2 with overlap syndrome 1 with Sjogren and 1 dermatomyositis) considered more than 5 B-lines as a positive result, and compared to HRCT, LUS presented a sensitivity of 73.58% and a specificity of 88.23% with positive and negative predictive values (PPV and NPV) of 95.12% and 51.72%, respectively [35]. Authors showed a good correlation of the two examinations; however, the number of patients included was small and LUS evaluated only 10 intercostal spaces bilaterally, according to the areas at higher prevalence of lung involvement in SSc at HRCT [35].

In 2014, two studies examined the use of LUS in RA patients [32,34]. The objective of the prospective study by Moazedi-Fuerst et al. was to test the frequency of LUS abnormalities in RA patients without clinical signs or symptoms of lung disease. Authors also enrolled 40 healthy volunteers (30% smokers) who, together with 64 RA patients (6% smokers), were evaluated by LUS. RA patients were also investigated by chest HRCT. Signs of lung involvement were detected in 28% of RA patients at LUS and 26.5% at HRCT, respectively. In only 10% of healthy controls, LUS was positive for lung involvement. Among RA patients with sonographic signs of lung disease, B-lines (>2 locations) were present in all patients, nodules in 12 and pleural fragmentation in 3 subjects. Interestingly, authors reported that among 18 RA subjects with LUS abnormalities, 16 presented equivalent changes at HRCT. One patient showed radiographic signs of lung involvement without abnormalities at LUS, and HRCT confirmed the presence of pulmonary abnormalities in 88% of patients with LUS signs of lung disease. Therefore, comparing the two instrumental examinations, authors reported a high sensitivity and specificity of LUS in the identification of pulmonary involvement (97.1% and 97.3%, respectively, with a PPV of 94.3% and a NPV of 98.6%), supporting LUS as a potential screening tool to assess lung involvement in asymptomatic RA patients [32]. Still, in 2014, Cogliati et al. analysed the accuracy of standard LUS in the evaluation of RA-ILD and the use of a pocket-size US device (PS-USD) as a screening tool. They evaluated the number of B-lines in 72 LIS score (28 anteriorly and 44 posteriorly) by standard LUS using a 5–2 MHz convex probe. The B-lines score was considered positive for lung involvement if >10. In this study, HRCT detected ILD in 13/39 patients, all positive for the rheumatoid factor. B-lines were >10 in 25/39 patients and the sensitivity and specificity of standard LUS versus HRCT were 92% and 56%, respectively. 29/39 were also examined by PS-USD, which, compared to HRCT, showed a sensitivity of 89% and a specificity of 50%. Data from this study support the use of LUS in clinical practice as a screening tool to identify when and which patients to undergo CT [34].

In the last years, other seven studies on LUS in RA patients have been conducted. Fotoh et al. performed a case–control study enrolling 140 RA patients (75 with ILD and 75 without). They demonstrated a higher level of serum KL-6 in patients with ILD. In addition, patients underwent LUS, and the number of B-lines was scored according to a semiquantitative system (0 or “regular” =< 5 B-lines, 1 or “mild” = from 6 to 15 B-lines, 2 or “moderate” = from 16 to 30 B-lines and 3 or “prominent” => 30 B-lines). The number of B-lines was significantly higher in RA patients with ILD and positively correlated with KL-6 and negatively with PFTs. In addition, they suggested the potential role of LUS score < 5.5 combined with serum KL-6 levels in the assessment of RA-ILD, correlating them with HRCT and disease severity [36]. Mena-Vázquez et al. confirmed the increased number of B-lines and pleural changes evaluated by LUS in RA patients with ILD compared to those without; however, they reported that in the eighth left posterior axillary space, the number of B-lines was similar in the two populations. In addition, regarding LUS score, authors reported that the detection of ≥10 B-lines evaluated in 72-space score was highly sensitive; however, the identification of 5.5 B-lines in eight intercostal spaces had a sensitivity of 62.2% and a specificity of 91.3% (with a PPV of 88.4% and a NPV of 69.5%), allowing a more specific and faster detection of ILD signs. In this population, the number of B-lines also positively correlated with ACPA and RA disease activity, and negatively with the diffusing capacity of the lungs for carbon monoxide (DLCO) [37].

Two studies conducted in 2022 suggested the key role of LUS in detecting which RA patients to address to HRCT, trying to avoid radiation exposure unless strictly necessary [38,39]. Di Carlo et al. enrolled 72 consecutive RA patients presenting at least one of the following clinical conditions: mild dyspnoea or velcro crackles at the physical examination; or at least two of the following risk factors: smoking habit, male sex, age older than 65 years, ACPA or rheumatoid factor. In these patients, LUS, PFTs, clinical examinations and chest HRCT were performed. B-lines positively correlated with HRCT findings (percentage of fibrotic changes) and negatively with PFTs values (DLCO and forced vital capacity). In addition, male RA patients showed a significantly increased number of B-lines when compared to females. Authors also suggested that the cut-off of nine B-lines may define the presence of a significant fibrosis at HRCT (sensitivity 70% and specificity 97.62%), also showing a potential role for LUS in the screening of RA patients without replacing HRCT with the sonographic examination [38]. A similar conclusion emerged from another study, investigating 74 RA patients with LUS [39]. ILD signs were revealed in 30/74 RA patients (40.5%) and in 3 controls (4.1%) (*p*-value < 0.001). ILD was detected by HRCT in 27 RA patients (36.4%). The concordance between the two examinations was 90% and in 5 RA patients LUS was false positive. In addition, authors confirmed a correlation between LUS changes, ACPA and rheumatoid factor [39].

Vermant et al. studied the anatomic distribution of lung involvement in RA-ILD in 75 patients by LUS in a total of 72 LIS. In this population, no correlation was found between LUS findings and gender, smoking habit, treatment or ACPA and rheumatoid factor. Sonographic abnormalities were detected in about half of enrolled patients, mostly represented by interstitial changes (presence of more than 5 B-lines), and the posterior sub-scapular regions as well as the anterior right mid-clavicular region were indicated as the hotspots for the presence of B-lines. In addition to these alterations, authors also reported other lung changes detected by LUS, represented by pleural abnormalities (in 45.3%), subpleural nodule (in 14.7%) and, rarely, pleura effusions (in 1.3%) [40].

Very recently, Otaola et al. evaluated 106 RA patients by HRCT and LUS, and this latter was considered positive for lung involvement when the number of B-lines was ≥5. Authors also investigated the presence of pleural alterations and the absence of A-lines (horizontal artifacts). LUS was positive in 49 patients and negative in 57. All patients were investigated by HRCT, which revealed the presence of ILD in 29/49 (59.2%) of patients with positive LUS and in 3/57 (5.3%) of those with negative LUS. Absence of ILD at HRCT was reported in 20/49 (40.8%) of patients with positive LUS and in 54 (94.7%) of negative LUS patients. In addition, authors showed that LUS was superior to radiography and PFTs [41]. Another recent study on 192 patients reported ILD at HRCT in 117 patients, of which 115 also presented a positive LUS evaluation. The overall agreement between LUS and HRCT was 65.6%. The sensitivity of LUS concerning HRCT was 98.3%, while a low specificity of 14.7% was reported. A moderate or severe interstitial involvement was shown in 86.5% of patients suspected for ILD at LUS, while pleural lines irregularity was detected in 93.3%. No concordance between the two examinations was reported in 64 cases, and in 97% LUS was false positive; in addition, this study suggested that a number of B-lines > 11.5 may predict ILD. Altogether, data coming from these two recent studies emphasized the role of LUS in the screening of RA patients, without suggesting that LUS can replace HRCT, which remains the gold standard in ILD assessment [42].

## 4. Discussion

Overall, these data highlight the possible key role of LUS in the detection of one of the most important extra-articular manifestations of RA. In fact, lung involvement in RA is associated with poor prognosis and increasing patient mortality and morbidity. For this reason, growing attention has been focused on its management and diagnosis. HRCT is the gold standard technique for ILD diagnosis. According to the enrolled population, prevalence and severity of ILD may considerably vary among RA patients, and some features are known to be associated with a higher risk of lung involvement, such as the above-mentioned male gender, smoking habit and positivity for rheumatoid factor and/or ACPA. However, in clinical practice, there is still no consensus on performing a screening HRCT in all patients with RA and with one or more risk factors, also considering its radiation exposure and cost. In addition, the fact that PFTs and clinical symptoms proved inadequate to detect early signs of lung involvement contributes to underdiagnosing ILD in RA patients. In the last decade, LUS has entered this panorama, confirming its key role in detecting ILD in patients with SSc [28]. Recently, using SSc as a model, the OMERACT US working group has performed the first step towards validation of LUS as an outcome measure technique [31]. Therefore, data coming from the scleroderma experience, as well as the recent consensus published by a multidisciplinary panel of LUS experts [44], encourage the research evaluating LUS role in other rheumatic diseases such as RA.

The reviewed studies strongly suggested LUSpotential role in the detection of ILD signs both in asymptomatic and symptomatic RA patients. The studies demonstrated the good diagnostic accuracy of B-lines and their correlation with HRCT findings; however, the methodology of this correlation was not uniform among all studies, and this point may influence the sensitivity and specificity values reported for LUS. In addition to B-lines, RA patients with ILD may present other abnormalities, particularly pleural nodules and fragmentation. Interestingly, compared to other rheumatic diseases, such as SSc and SLE, patients with RA seem to present more frequently US findings other than B lines as pleural nodes [33], probably reflecting the different radiological patterns in these diseases.

Therefore, the results coming from the revised studies indicate that LUS may represent a novel, non-invasive, and non-ionizing imaging method in the evaluation of lungs in RA patients. However, LUS cannot replace HRCT, which still represents the gold standard examination for ILD diagnosis. In addition, it is important to remark that RA may potentially affect all compartments of the pulmonary system and involvement other than ILD may be present, like pleurisy, airways disease, lung rheumatoid nodules and increased risk of infections and cancer. LUS may represent a screening tool of lung involvement, only considering ILD or pleural involvement as all the other conditions escape US evaluation, necessarily requiring HRCT when they are suspected. Furthermore, patients with RA may present pulmonary comorbidities such as heart failure, asthma, pulmonary edema or pulmonary involvement due to smoking habits. The presence of one of these conditions was considered an exclusion criterion in the revised studies. In fact, LUS abnormalities such as B-lines or pleural alterations are unspecific signs of an “interstitial syndrome”. In fact, it is known that B-lines may be present in all conditions causing interstitial involvement, as pulmonary edema due to different reasons or interstitial pneumonia. In addition, when B-lines are multiple and focal in a normal lung, this may indicate a pneumonia, a pulmonary contusion or even a pleural disease or a neoplasia [45]. The concomitant presence of multiple B-lines and pleural alterations in patients with dyspnoea may also indicate an acute respiratory distress syndrome (ARDS) [46]. These data highlight the lack of specificity of LUS and of its inability to evaluate the whole parenchyma [47,48], representing its main limitations. Therefore, LUS cannot replace HRCT in RA patients, as the radiological examination allows the evaluation of all pulmonary structures, a differential diagnosis of ILD with other conditions such as damage due to smoking and the detection of possible concomitant conditions such as infection and cancer.

However, despite its limitations, the preliminary results of the herein revised studies in RA and the experience coming from other rheumatic disease, such as SSc where LUS has proven to be a valid screening test also correlating with ILD extension at HRCT [49], emphasize the promising role of LUS in the evaluation of RA patients. In addition, the low cost and non-invasive nature of this examination allow for the evaluation of a larger number of patients and more frequently during the follow-up, providing information that, together with the clinical evaluation and the presence of risk factors for ILD, may drive the clinician to evaluate the need of HRCT, both at baseline and during the disease course. LUS could help the early identification of lung involvement, extending the evaluation even to patients who apparently do not present clinical or disease-related risk factors, but who may present this fearful disease complication. Therefore, altogether, the reported studies support the use of LUS in the assessment of ILD in RA patients. However, future research is widely needed to validate this technique as a screening tool of ILD in RA, and in this context, it will also be important to assess the extension of ILD at HRCT in order to evaluate the sensitivity and specificity of LUS. The validation of this technique as a screening tool could place LUS in the patient’s baseline assessment.

## 5. Conclusions

LUS is a radiation-free, non-invasive and low-cost examination that may be considered in RA patients to evaluate the presence of signs indicative of an “interstitial involvement”, and the revised studies reported a concordance of LUS findings with HRCT, which represents the gold standard technique for ILD diagnosis. These data open new perspectives in the management of RA-ILD, in which LUS may represent a useful preliminary measure allowing, together with the clinical evaluation and the assessment of risk factors, the detection of which patients need a HRCT evaluation, even if asymptomatic. Further large-scale, multi-center and prospective studies are needed to validate LUS in RA patients.

## Figures and Tables

**Table 1:** Reviewed studies and their principal characteristics.

References	Number of Enrolled Patients	Excluded Conditions	Probe	Number of Areas Evaluated by LUS	Alteration Assessed
Moazedi-Fuerst F et al., 2015 [33]	- 45 consecutive patients (25 RA, 14 SSc, 6 SLE) - 40 healthy volunteers	Acute pulmonary complaints	- Convex 3.5 MHz transducer - Linear probe (for the pleura)	18 regions total	- B-lines - Pleural nodes - Pleural thickness
Aghdashi M et al., 2013 [35]	- 31 consecutive patients (19 SSc, 8 RA, 2 overlap syndrome, 1 Sjogren, 1 dermatomyositis)	-Pulmonary cancer - Suspicion of lymphangitis carcinomatosa - Other causes of interstitial involvement (example: heart failure, asthma, pulmonary edema and history of smoking)	- 7–10 MHz linear multi-frequency transducer	10 intercostals space bilaterally	B-lines (more than 5 B-lines considered positive)
Moazedi-Fuerst et al., 2014 [32]	- 64 RA patients - 40 healthy volunteers	- Known lung diseases, signs of infection - Present or previous dyspnoea, coughing or thoracic pain - Abnormal results from previous chest radiographs	- Convex 3.5 MHz transducer (for the parenchyma) - Linear probe (for the pleural assessment)	18 regions total	- B-lines - Pleural noduli - Pleural line
Cogliati et al., 2014 [34]	- 39 RA patients	- Diagnosis of pneumonia in the last month - Pleural effusion determining parenchyma atelectasis - Cardiac disease as possible cause of heart failure (including patients with moderate to severe mitral or aortic valve disease, left atrial or ventricular dilatation, ejection fraction <50%, diastolic dysfunction)	- 5–2 MHz convex probe - PS-USD with a phased array transducer (1.7–3.8 MHz)	72 lung intercostal spaces	- B-lines
Fotoh DS et al., 2021 [36]	- 140 RA patients	- Conditions causing CT exacerbation (pneumonia, recent respiratory infections, presence of multiple autoimmune diseases, heart failure, lung surgery) - History of asthma or COPD - Lung malignancy - Renal failure - Pregnancy	Linear transducer 4–13 MHz	14 lung intercostal spaces (50 scanning sites)	- B-lines
Mena-Vázquez et al., 2021 [37]	- 71 RA patients	- Inflammatory or rheumatic diseases other than RA (except secondary Sjögren syndrome) - Infection - Primary PH - Heart disease - Known exposure to environmental fibrosing agents - Pregnancy	- 2.5–3.5 MHz transducer	72 intercostal spaces	- B-lines - Pleural abnormalities (increased in thickness, fragmentation, loss of the normal linear pattern)
Di Carlo et al., 2022 [38]	- 72 RA patients	- Positive history of fibrosing lung diseases other than suspected RA-ILD - Suspected pulmonary toxicity from methotrexate - Other lung conditions (severe congenital or acquired thoracic deformities, previous lung surgery, recent or current history of low respiratory infections) - Heart failure	- 4–13 MHz linear probe	14 lung intercostal spaces	- B-lines
Gutierrez M et al., 2022 [39]	- 74 RA patients - 74 healthy control	- Previous history of acute or chronic pulmonary diseases (example: asthma, COPD, pulmonary edema due to heart failure) - Recent or current history of low respiratory tract infections - Previous lung surgery	14 lung intercostal spaces bilateral	- 5–13 or 4–12 MHz linear transducers	- B-lines
Verman M et al., 2023 [40]	- 75 RA patients	_	72 lung intercostal spaces	- Curved 3.5 MHz array probe	- B-lines - Pleural abnormalities - Subpleural nodules - Pleura effusions
Otaola M et al., 2024 [41]	- 106 RA patients	- History of any disease potentially affecting LUS evaluation (pneumonia in the last month, presence of pleural effusion, cardiac disease, lung cancer)	14 chest areas	- 1–8 MHz convex probe	- B-lines - Pleural line change - Absence of A-lines
Santos-Moreno P et al., 2024 [42]	- 192 RA patients	- Pregnancy - Pneumonia in the last month - COPD - Pleural effusion (moderate or severe) - Atelectasis - ILD due to CTDs - History of COVID-19 infection	All intercostal spaces, divided in anterior, posterior and lateral area on each side	- 2–5-MHz convex transducer	- B-lines - Irregularity of pleural line

Abbreviations: RA = rheumatoid arthritis; ILD = interstitial lung disease; LUS = lung ultrasound; SSc = systemic sclerosis; SLE = systemic lupus erythematosus; COPD = chronic obstructive pulmonary disease; CTDs = connective tissue diseases; COVID-19 = coronavirus disease 2019.
